# What’s next: using infectious disease mathematical modelling to address health disparities

**DOI:** 10.1093/ije/dyad180

**Published:** 2023-12-25

**Authors:** Danielle M Richard, Marc Lipsitch

**Affiliations:** Center for Forecasting and Outbreak Analytics, Centers for Disease Control and Prevention, Atlanta, GA, USA; Center for Forecasting and Outbreak Analytics, Centers for Disease Control and Prevention, Atlanta, GA, USA; Department of Epidemiology, Harvard T.H. Chan School of Public Health, Boston, MA, USA

**Keywords:** Infectious disease epidemiology, mathematical modelling, social determinants of health, social epidemiology, health equity

Before and during the COVID-19 pandemic, an individual’s age and race/ethnicity have been highly predictive of their risk of infectious diseases and their health consequences. Disparities were evidenced in COVID-19 incidence rates and in hospitalization, severity and mortality metrics in the USA^1^ and in other countries.^2^^,^^3^ Identifying these disparate outcomes associated with demographic variables is valuable mainly if it prompts investigation into what mechanisms generate the disparities and inform how they can be reduced.^4^ A prominent report from the UK succinctly outlined that social determinants such as occupation, household characteristics, surrounding population density, urbanicity and social deprivation were all associated with increased risk of COVID-19 infection.^3^ Others have noted that social determinants can play a role in all stages of an outbreak, providing pathways for unequal exposure, transmission, susceptibility and treatment that produce and escalate disparities in health outcomes.^5^

In addition, the use of mathematical and computational infectious disease transmission models expanded during the COVID-19 pandemic. Researchers and decision makers used infectious disease models to estimate key outbreak parameters, inform how policy changes could affect the ongoing outbreak and predict what would happen next in various settings. Although social determinants were considered on occasion, few infectious disease transmission models were used to answer questions on the structural role of social determinants in the proliferation of the COVID-19 outbreak.^6^ To understand the origin of inequities in future pandemics and inform policies to reduce them, it is important to incorporate these determinants not only in data-gathering efforts, but also in model structure and analysis, as is appropriate for a given research question.

We aim to outline how infectious disease transmission modelling has been used to identify the mechanisms that create health disparities, to describe how mechanisms can change over the course of an epidemic and to propose policy recommendations that are informed by epidemiological principles. Although this review is not a systematic analysis of all existing literature, we aim to inform the reader on the current state of the literature and on what is missing. We describe examples of infectious disease models that address health equity questions that fall into three categories: deterministic compartmental models, agent-based models and extensions to deterministic models that incorporate mobility data. We conclude by providing recommendations for how we can work to address what is missing in the infectious disease modelling field, so that we can be better prepared moving forward, for all respiratory diseases and not just COVID-19.

## Deterministic compartmental models

Simple, equation-based infectious disease models structure the population into categories (compartments), most fundamentally those of Susceptible–Exposed–Infectious–Recovered (SEIR). SEIR models that have addressed health equity questions have considered methods to mechanistically incorporate social determinants into the structure of their models by parameterizing contact, susceptibility and other determinants of transmission separately for different social groups. A study by Menkir and colleagues is one example. The research team used income quintile-specific values for disease-related parameters such as disease transmission rates, case-fatality rates and vaccine coverage in a susceptible–infectious–recovered (SIR) model to examine the impact of varying vaccination strategies. By incorporating parameters stratified by income level, the researchers were able to assess the extent of differential infectious disease outbreaks spread in distinct subgroups of the population. Of note, findings indicated that, for lower-income quintiles, maximum prevalence was reached earlier than for other income groups. Further, higher within-quintile transmission translated into higher infection peaks, and higher vaccination coverage yielded the greatest benefit to the lowest-income quintiles.^7^ The effort to construct such models also pointed to limitations in the availability of data to estimate stratum-specific transmission and severity parameters, highlighting the close dependence of models on high-quality data stratified by social determinants.

Other studies have addressed other questions of health equity by structuring transmission models to incorporate specific determinants. One team incorporated age-structured models for two social groups with high and low transmission into their SEIR model and used social contact data to parameterize contact rates for different social groups. This study varied parameters for contact intensity between the two groups and for susceptibility to infection, and derived predictions on how interventions can impact inequalities. Perhaps counter-intuitively, introduction of a vaccine with equal coverage in interacting high- and low-risk groups led to a greater relative inequality in disease incidence between the groups than in the absence of vaccination; this occurs because a given level of coverage comes closer to eliminating transmission in the low-risk group with lower transmission. This theoretical study did not specify explicitly the reason for the high- and low-contact groups, but rather assumed their existence and derived the consequences. This method could be extended to include particular social determinants, and still provides an example of thinking about inequalities in model production.^8^ To quantify how social exposure to infection varies across race and ethnicity, Ma *et al.* created separate sets of compartments for a selection of race and ethnicity groups, and used a social contact matrix estimated from housing segregation data to account for between-racial-group mixing. Findings indicated that the herd immunity threshold was reached after the cumulative incidence disproportionately increased in certain minority groups and that, even if the same groups continued to have higher contact rates over time, disparities in incidence could shrink and even reverse over time as a result of higher immunity in such communities.^9^ Such approaches described thus far expand our analytic capacity and allow researchers to consider mechanisms by which disparities may exist, how they depend on the phase of the epidemic and how different interventions, such as vaccinations, may ameliorate or inflate existing disparities. Improving the quality and level of detail of data to parameterize these models would enhance their usefulness.

There are also examples of models that have been used to examine outbreaks of a disease in specific vulnerable populations, such as those experiencing homelessness, or in specific settings, such as correctional facilities or nursing homes. To better understand the role of correctional facilities in the proliferation of disease, one team utilized compartmental modelling of infections within and between correctional facilities and the surrounding community for people who are incarcerated, correctional staff and surrounding community. The team found that, when an infectious disease outbreak occurs in a correctional facility, the outbreak gets reflected and magnified back to the surrounding community. Additional analyses were used to inform critical factors in determining the magnitude of an outbreak, and how movement of staff and people who are incarcerated can play a role.^10^ In the UK, Lewer and colleagues used SEIR models to understand how homelessness policies, such as hotel accommodations or infection control in homeless settings, affected overall case counts, deaths and hospital admissions in England. Findings indicated that lifting preventive measures led to large increases in cases and deaths for people experiencing homelessness.^11^ Modelling efforts such as these not only help decision makers to better understand the marked disparities in infectious disease outbreaks, but also can better represent infectious disease outbreaks by incorporating setting-specific transmission parameters. This merits not only separate modelling efforts, but also an effort to better understand how outbreaks within a subset of the population that is more vulnerable can extend to the larger population.

## Agent-based models

Several studies have employed agent-based models to demonstrate how population structure and transmission dynamics help explain observed inequities. To better understand the cause of differences in influenza across census tracts of varying poverty levels, Kumar and colleagues used an agent-based model to simulate influenza attack rates across census tracts and with varying neighbourhood contact rates and income levels. Census tracts with higher poverty had earlier and steeper increases in infection rates—a pattern observed in both real-world data and in model output. Further, the team estimated that including population structure and population mixing in simulation modelling accounted for 33% of the observed inequality in infection prevalence between census tracts of high and low poverty levels.^12^ To assess the relationship between influenza burden and social deprivation, Hyder and Leung used a spatially explicit model of Montreal that incorporated household composition as well as other social determinants with spatial arrangements of neighbourhoods. The authors found that heterogeneity of neighbourhood composition and spatial arrangement of neighbourhoods contributed to the observed relationship between influenza burden and social deprivation.^13^

There are also examples of agent-based models that are intended to guide policy recommendations. Focusing on infection control in nursing homes, another team used an agent-based model of SARS-CoV-2 transmission to highlight how different non-pharmaceutical interventions would affect disease incidence. The team found that daily antigen testing, compared with other interventions such as cohorting or staffing interventions, reduced the cumulative incidence the most, whereas combining screening testing with resident cohorting and immunity-based staffing interventions reduced the cumulative incidence even more.^14^ In another example, Nande *et al.* used a network model incorporating within- and between-household mixing to assess the impact of evictions on household transmission of COVID-19, accounting for evictions that led to households moving in with other households. Findings indicated that evictions would increase COVID-19 cases across the population and that a simulated increase in the eviction rate of 1% per month resulted in an infection level that was ∼4% higher than baseline. Further, evictions were predicted in the simulations to increase the disparity of infection prevalence between low- and high-socio-economic status (SES) neighbourhoods, where the risk of infection increased more for the low-SES neighbourhoods. This study exemplifies how an infectious disease model can be adapted to consider the impact of a policy change on vulnerable populations and inform decision makers with estimates of policy impacts.^15^ Given sufficient data to constrain their assumptions, agent-based models and simulation studies such as these can be powerful tools to consider how policies and changing epidemic quantities can affect outbreaks in specific settings or subpopulations.

## Using mobility data in deterministic models

In recent years, the field of infectious disease modelling has started incorporating mobility data into infectious disease models to inform parameter estimation and as a proxy for social characteristics. One team incorporated SafeGraph mobility data into an underlying SEIR model to estimate the effects of specific reopening strategies and to predict infection rates in areas with different demographic compositions. The authors found that lower-income areas did not have as much reduced mobility and that larger proportions of individuals from lower-income areas were infected at specific locations due to higher contact density; the role of higher contact density in promoting transmission was a model assumption and the fact that lower-income individuals had more exposure to high-density locations thereby offered a hypothesis to explain the higher incidence of infection among such individuals.^16^

Another way to incorporate individual contacts into a model is by using contact survey data. Many other countries have contact survey data that are derived from POLYMOD—a paper-diary survey with data for >7000 participants in eight European countries.^17^^,^^18^ Because the USA does not have readily available and nationally representative contact data, one research team utilized an egocentric exponential random graph model based on POLYMOD data in combination with an SEIR model to assess inequalities in influenza transmission in the USA. The researchers proposed five potential immediate drivers of influenza transmission inequities: social contact differences, low vaccine uptake, high susceptibility to infection, low healthcare utilization and low sickness absenteeism. Findings indicated that, when SES-specific parameters were used, all five factors were associated with an increase in burden for the group of people with low SES.^19^ Notably, there are contextual reasons for why each of these factors may be seen more often in people with low SES. Describing this is beyond the scope of this discussion.

Although mobility data and other data sources from novel technologies are proving to be powerful in some applications, they also come with inherent biases that must be recognized. More research is needed to identify how applicable mobility data is at larger spatial scales, where there is considerable within-population heterogeneity in mixing patterns and other social drivers,^20^ and how changes in contact patterns may be proxied by mobility data.

## Recommendations for embedding health equity principles into infectious disease modelling

Although there is ongoing research and interest in the use of infectious disease modelling to address health equity concerns,^21^^,^^22^ there is much to be done. First, more studies that incorporate social determinants into compartmental, network and agent-based models are needed. As discussed, this can be accomplished by tracking outcomes based on social determinants and by incorporating social determinants into model processes (transmission, outcomes of infection, etc.). Further, improved models for specific settings such as shelters, schools, prisons and jails, healthcare settings and nursing homes and other congregate settings are needed to better prepare us for future emerging infectious disease threats. Researchers can more effectively assess health disparities by creating models for vulnerable populations that incorporate the effects of policies on transmission. Our review demonstrates that there are quality examples of using infectious disease modelling to better understand health disparities and that we must continue to prioritize such models as the burden of infectious disease outbreaks continues to fall on disadvantaged groups.

Second, a renewed focus on gathering representative incidence and seroprevalence data in emerging outbreaks is needed. Public health surveillance in the USA remains less likely, for several reasons, to include individuals of lower SES. For example, those of lower SES are less likely to receive healthcare and therefore be included in local surveillance efforts. Some people of colour and people with diverse gender identities are more likely to experience stigma or racism in healthcare and may be less likely to seek healthcare when needed.^23^ There are ongoing activities across the USA public health landscape to improve upon the existing surveillance systems to reduce these biases. Even so, accounting for and, where possible, adjusting for existing surveillance biases in analytics is critical, as is properly communicating our analytic findings with the correct caveats to interpretation. To further address these biases, random sampling of the prevalence and incidence of infection can provide key data to answer questions about existing disparities. Without these data, it is difficult to identify where in the cascade of prevention and care inequities occur because higher rates of disease are indistinguishable from higher rates of infection in vulnerable populations. The benefit of random sampling can be seen in the Real-time Assessment of Community Transmission-1 (REACT-1) study, conducted in the UK.^24^^,^^25^

In newly emerging outbreaks, such as in the early days of COVID-19, gathering detailed data on proximal, mechanistic social risk factors for infection or a history of infection (seropositivity) can directly inform efforts to equitably reduce risk. Further, re-collecting and reanalysing these data iteratively over the course of an outbreak are also critical. In particular, when racial/ethnic minorities and/or those of lower SES are harder hit, as is often the case, choice of interventions depends on knowing the contribution of occupational exposure, household crowding, rurality or inability to isolate in disparities in exposure and the contribution of access to care or other causes of disparities in outcome. Such data, which are gathered in routine serologic studies such as the US National Health and Nutrition Examination Survey and sporadically in other studies, are much more helpful than simple race and ethnicity data in informing solutions.^26^ Gathering these proximate risk factors is consistent with the increasingly accepted concept that race is not an exposure variable and should not be treated as such in modelling. Rather, a race variable can serve as a proxy for social determinants, as well as for lived experiences of racism and stigmatization.^4^^,^^27^ Knowing what specific mechanistic risk factors are associated with the outcome is necessary if the research is to inform solutions, rather than simply document disparities.^3^ A better understanding of how social determinants can change over the course of an outbreak for susceptible, infected and recovered populations can also be illuminating. If the epidemic is primarily in one subgroup of the population at first, but then is predominantly in other subgroups as the outbreak continues, the roles of these subgroups in driving transmission can be inferred and serve as a prompt for intervention.^28^ Conversely, understanding how disparities will evolve over the course of an epidemic even without interventions can provide appropriate baselines for assessing how well interventions work to curb disparities.^9^

Third, we must identify epidemic quantities of interest for subgroups of populations. A key limitation across many of the aforementioned studies was that there was little to no availability of data for key epidemic quantities in specific settings of interest or for specific subsets of the population. Improving our case data infrastructure so that we have data for social determinants would allow us to calculate parameters stratified by social determinants.

Towards this end, micro-scale studies of transmission^29^ and contact patterns^18^ as a function of household size and density are needed to provide an empirical basis for the detailed structure and parameterization of these models, e.g. to decide how transmission differs in large and small households, or as a function of density in public places. Given the uptick in interest in ventilation as an intervention to reduce transmission, better understanding of how transmission rates vary across settings could have the additional benefit of informing investments in ventilation to make them more effective and more equitable.

Lastly, we need country-specific contact surveys that are stratified, as appropriate for the specific population, by race and ethnicity and by social determinants. In the USA, although researchers have been able to adapt the POLYMOD contact study to their needs, investing in a comprehensive USA-based contact survey with a priority placed on obtaining data for participants’ social determinants could greatly expand the possibilities for mathematical modelling there.^7^ As noted above, stratifying these data by household sizes and/or by setting would also be beneficial.

Although the fields of infectious disease modelling and social epidemiology have expanded greatly in the past several decades, there is much to be done to formally embed epidemiologic principles into mathematical modelling methods. As the field of infectious disease mathematical modelling continues to expand and adapt to new and emerging public health threats, it is critical that we prioritize the development of methods to mechanistically account for social determinants and produce models that inform policies that can address the root causes of health disparities.

## Ethics approval

Ethics approval is not required for this study because human patients were not involved.

## Data Availability

No new data were generated or analysed in support of this research.
